# Epidemiology and pain in elementary school-aged players: a survey of Japanese badminton players participating in the national tournament

**DOI:** 10.1038/s41598-021-85937-5

**Published:** 2021-03-19

**Authors:** Xiao Zhou, Kazuhiro Imai, Xiao-Xuan Liu, Eiji Watanabe

**Affiliations:** 1 Department of Life Sciences, Graduate School of Arts and Sciences, The University of Tokyo, 3-8-1, Komaba, Meguro-ku, Tokyo, 153-8902 Japan; 2 Institute of Sport, Senshu University, Kawasaki, Kanagawa Japan

**Keywords:** Preventive medicine, Risk factors, Epidemiology

## Abstract

Pain is common in athletes which should be well managed. To identify risk factors for shoulder pain, and the association between shoulder pain, lower back pain and knee pain among elementary school-aged badminton players, we conducted a cross-sectional study to collect data of the past year among 611 elementary school age (7–12 years old) badminton players belonging to the Japan Schoolchildren Badminton Federation using a questionnaire. Odds ratio (OR) and 95% confidence interval (CI) were estimated by multivariate logistic regression analysis. The overall incidence rate of shoulder injuries, lower back injuries and knee injuries was 0.38 injuries per 1000 h of badminton training. Players with training time per day > 2.5 h were 2.64 times (95% CI 1.03–6.78, *p* = 0.043) more likely to sustain shoulder pain than those with training time per day ≤ 2.5 h. A significant association was revealed between shoulder pain and knee pain as well as between lower back pain and knee pain as training hours per day > 2.5 h. Moreover, lower back pain was significantly associated with shoulder pain independent of training hours per day (≤ 2.5 h: *p* = 0.001; > 2.5 h: *p* < 0.001). These findings indicate that training time per day is risk factor, and shoulder pain, lower back pain and knee pain were associated with each other in elementary school-aged badminton players.

## Introduction

Shoulder pain and injuries are common problems in badminton players. Epidemiological studies on badminton reported incidences of shoulder injuries of approximately 1.4–19.0% in badminton players aged 7–57 years^1–3^. For injury incidence rate per 1000 h of shoulder injuries, 0.30 injuries per 1000 h in badminton players, including 0.33 in elite senior badminton players, 0.50 in elite junior badminton players and 0.12 in potential badminton players. Shoulder pain was caused by subacromial impingement, instability or scapulothoracic dyskinesia^4^ that approximately 27.0–52.6% of badminton players experienced shoulder pain^2,5,6^. Shoulder pain would change overhead motion performance negatively and affect activities of daily living such as sleeping disturbance^7,8^. However, more than one-third of badminton players with shoulder pain continued to play, which might lead to shoulder injuries^7^. It is crucial to assuring long-term, pain/injury free participation, especially for youth players. Therefore, to identify risk factors of shoulder pain could be a better way to implement early intervention for preventing shoulder pain.

Literatures reported increased age^9–11^, female gender^10^, increased badminton hours weekly^2^, badminton match^10^ and low badminton level^2^ were risk factors for badminton injuries. However, as far as we have searched, there are currently no studies on risk factors of shoulder pain in badminton players. Studies on other overhead motion sports, e.g., baseball, reported increased age^12,13^, lower height^12^, hard training intensity^13^ and longer training hours weekly^14^ were risk factors for shoulder pain.

The forehand overhead stroke motion of badminton, referred to as a kinetic chain allows energy generated by ground reaction, and activity of lower limbs and trunk muscles to be transferred to the upper limbs^15,16^. A breakage in any of the kinetic chain may cause pain or injuries^17,18^. Lower back pain and knee pain were demonstrated significant association with shoulder pain in youth baseball players^13^. A study of overhead players which included badminton players stated a significant association between shoulder pain and back or lower limb pain, however, it was unspecific, and the number of badminton players was small^18^.

Biological changes resulting from injuries lead to sports incapacity (time loss) that impact sports performance and livelihood directly^19^. Evaluation of the entire body is crucial to examining injured players^11^ and preventing badminton injury. Nevertheless, risk factors for shoulder pain associated with badminton, and association between shoulder pain, lower back pain and knee pain among badminton players are not well understood. Therefore, an epidemiological study with a large number is essential to assure safe participation for badminton players. The purpose of this study was to identify risk factors for shoulder pain, and the association between shoulder pain, lower back pain and knee pain among elementary school-aged badminton players so that injury prevention and intervention can be implemented as early as possible. Based on the previous literatures, we hypothesized that lower height and training hours per week are risk factors for shoulder pain, and there is an association of shoulder pain, lower back pain and knee pain.

## Methods

This study was a collaboration between the Graduate school of Arts and Sciences, the University of Tokyo, Institute of Sport, Senshu University, and Japan Schoolchildren Badminton Federation. Participants in this study were elementary school-aged badminton players (aged 7–12 years) belonging to the Japan Schoolchildren Badminton Federation and participating in the national tournament. Data was collected by a self-reported questionnaire from 611 badminton players (aged 7–12 years, 260 males and 351 females) who were consented by their guardian in May–August 2019. This study was reviewed and approved by Ethical Committee of the Graduate school of Arts and Sciences, the University of Tokyo, Japan (Notification Number 602-2 July 26, 2018). The study protocol was performed in compliance with the tenets of the Declaration of Helsinki. The questionnaire asked for information including gender, age, height, weight, badminton experience, training hours per day, training days weekly, warm-up, cool down, and pain or injury histories associated with badminton. Adequate training volumes not only decrease non-contact, soft-tissue injury risk, but also boost physical qualities^20^, thus, we used training hours per day and training days per week to collect training volumes. Training hour (athlete exposure hour) was defined as time of badminton skills or physical condition training under supervision of the coach, but warm-up and cool-down time were excluded. Pains and injuries were specifically reported regarding anatomical sites. The anatomical sites were presented using a picture showing body parts including face, neck, shoulder, elbow, hand, lower back, groin, thigh, knee, Achilles tendon, ankle, foot and other. A pain was defined as any physical painful complaint or discomfort with sustained badminton capacity. An injury was defined as any physical complaint sustained during training or match play resulting in one or more of the three judgement criteria as follows: (1) have to stop the current training or match immediately, (2) absence from subsequent training or matches, and (3) require medical attention with time loss injuries. Injuries were categorized as traumatic injuries which has acute onset, and gradual-onset injuries defined by chronic physical complaint without traumatic injuries. Excluded missing data (n = 101), 510 badminton players, including 217 male and 293 female badminton players were included in this study.

First, distributions of pains and injuries in shoulder, lower back and knee among the 510 badminton players were described. The injury rates of shoulder, lower back and knee injuries were calculated as per 1000 athlete-hours of exposures, with 95% confidence interval (CI) using Poisson distribution. The injury rate of per 1000 athlete-hours of exposures in the badminton period of the past year is calculated as follows:

$${\text{Injury rate per 1}}000\;{\text{athlete}} - {\text{hours of exposures}} = \, [\sum ({\text{No}}.{\text{ of injuries}})/\sum \{ ({\text{No}}.{\text{ of participants}}) \times ({\text{hours of training}})\} ] \times {1},000.$$

Then, according to the definition of injury and pain, 460 badminton players without badminton-related injuries were screened for further pain analysis. All the 460 badminton players included 194 males (7–8 year-old: 35 males; 9–10 year-old: 81 males, 11–12 year-old: 78 males; mean ± SD, age: 9.9 ± 1.4 years, height: 144.1 ± 7.0 cm, weight: 33.5 ± 6.5 kg, BMI: 16.0 ± 2.4, badminton experience: 2.5 ± 1.6 years), and 266 females (7–8 year-old: 41 females; 9–10 year-old: 109 females, 11–12 year-old: 116 females; mean ± SD, age: 10.0 ± 1.3 years, height: 142.6 ± 5.9 cm, weight: 32.7 ± 6.4 kg, BMI: 16.0 ± 2.8, badminton experience: 2.5 ± 1.5 years).

Medians with interquartile range (IQR) were adopted to present continuous variables which were categorized according to the distribution, and categorical variables were shown in numbers and percentage. The variables including gender, age, body mass index (BMI, calculated by height and weight values of the participants), badminton experience, training hours per day, training days per week, total hours per week, doing warm-up and cool down or not were considered potential confounding factors.

### Statistical analysis

We operated crude analysis and multivariate logistic regression analysis which included all the variables in the model to examine the association between shoulder pain, lower back pain and knee pain, respectively. Next, adjusted odds ratio and 95% confidence intervals were analyzed by multivariate logistic regression analysis to detect association of all the variables with shoulder pain. Furthermore, the participants were stratified into two groups by the risk factor of shoulder pain. The association of shoulder pain, lower back pain and knee pain in two groups were investigated using multivariate logistic regression analysis, respectively. Odds ratio (OR) with 95% confidence intervals (CI) was performed for results of the multivariate logistic regression analysis model.

Variables were divided into categories as following: gender (male or female), age (7–8, 9–10 or 11–12 years), badminton experience (< 1, 1– < 3 or ≥ 3 years), training hours per day (≤ 2.5, or > 2.5 h), training days per week (≤ 3, 4–5, or 6–7 days), total hours per week (≤ 11, or > 11 h), warm-up (yes or no) and cool down (yes or no).

We calculated the sample size by referring to the basis of prevalence of badminton injuries reported among youth competitive badminton players^21^. For logistic regression analyses, a sample size of 360 participants would be needed to examine statistical significance depending on an 90% power and an alpha level of 0.05 calculation (assumptive incidence rate = 40%, odds ratio = 2.0). If we assume a dropout rate of about 20%, 450 players would have to be recruited.

## Results

### Epidemiology of shoulder, lower back and knee

**Figure 1:**
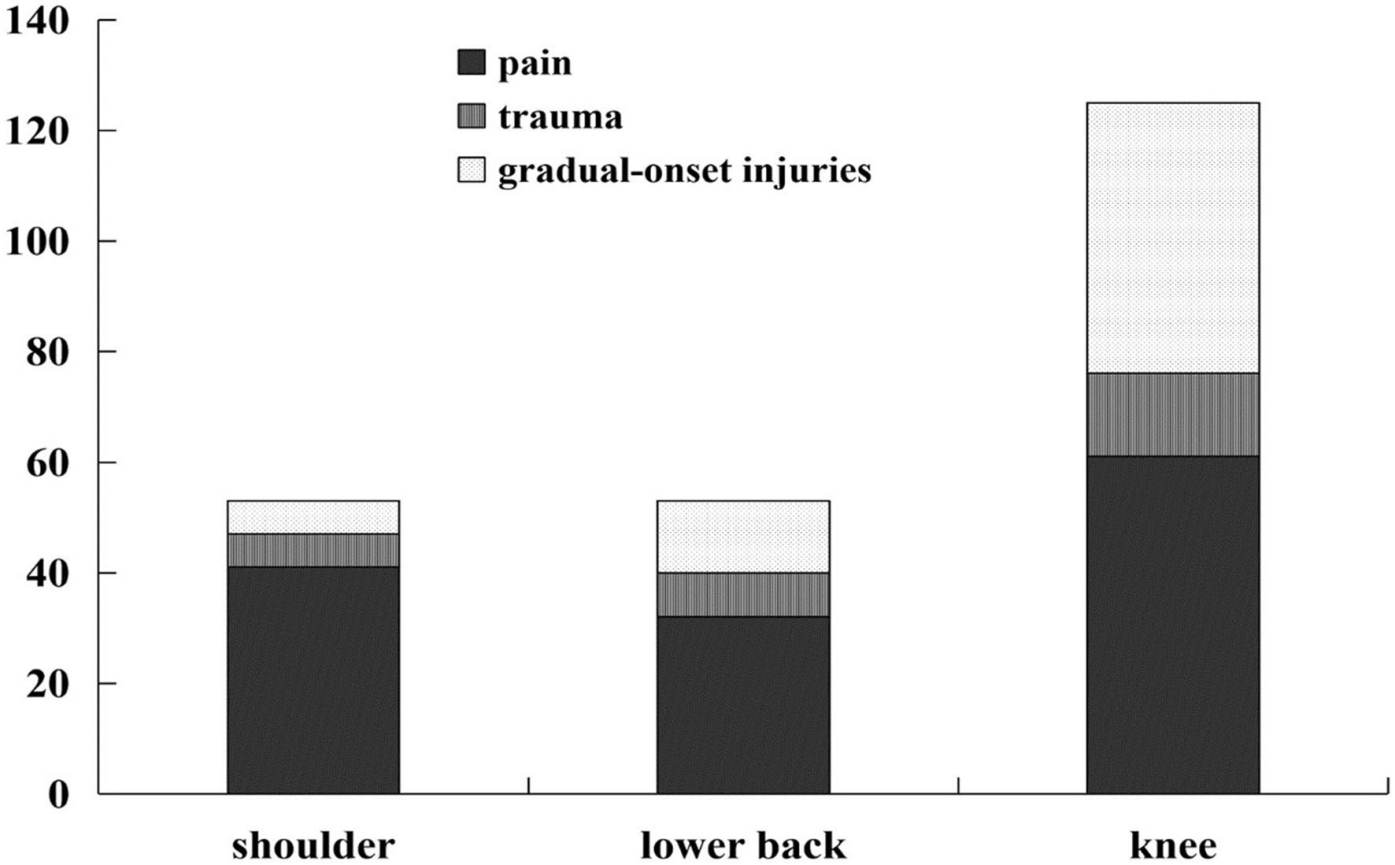
The distributions of pain, trauma, and gradual-onset injuries in shoulder, lower back and knee.

**Figure 2:**
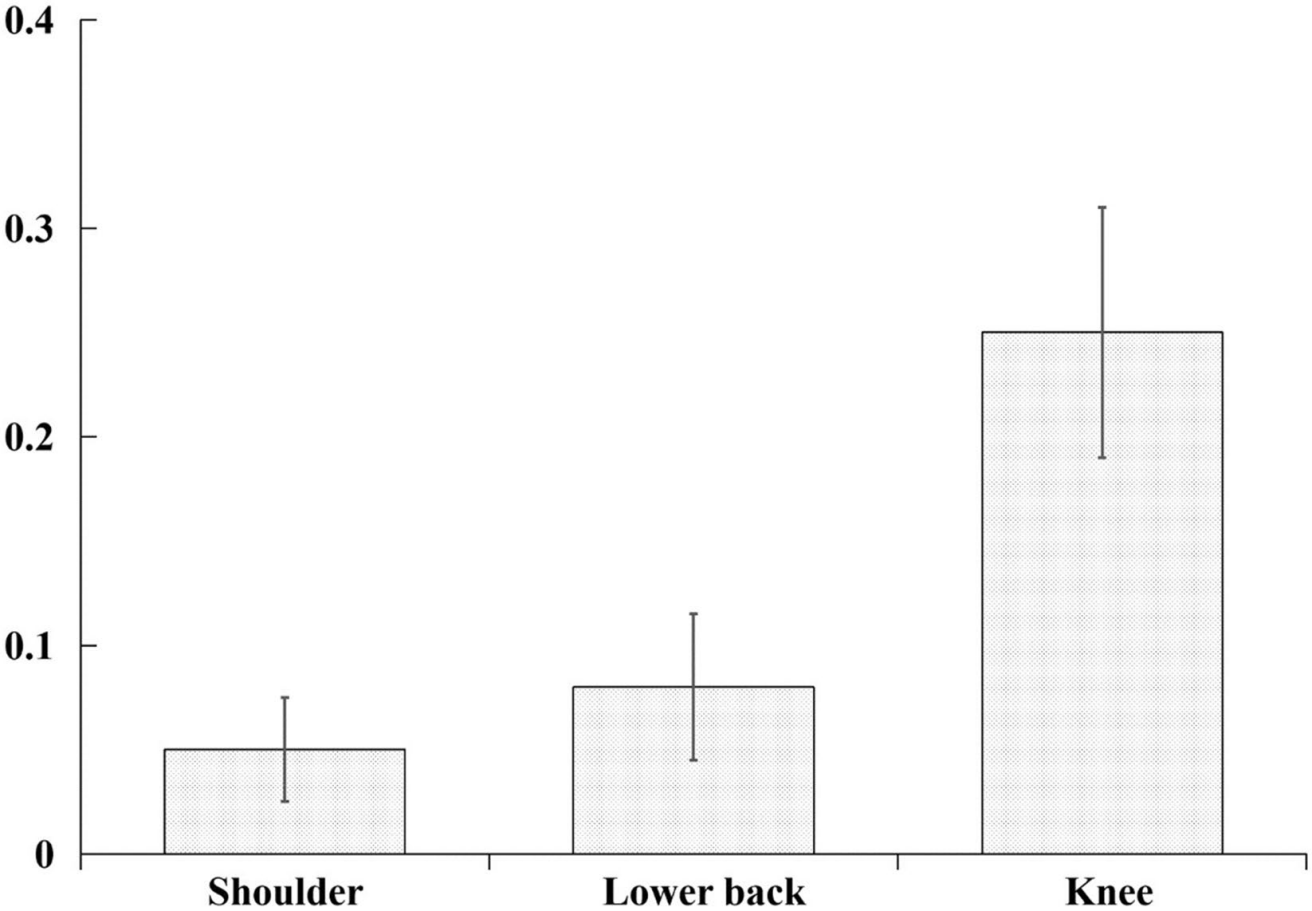
Injury rate per 1000 athlete-hours of exposure in shoulder, lower back and knee. The error bars represent a 95% confidence interval from the mean.

The distributions of pains, traumatic injuries and gradual-onset injuries in shoulder, lower back and knee among all the 510 elementary school age badminton players are shown in Fig. 1. In shoulder, 41 cases of pain, 6 cases of traumatic injury and 6 cases of gradual-onset injury were reported. In lower back, 32 cases of pain, 8 cases of traumatic injury and 13 cases of gradual-onset injury were reported and in knee, 61 cases of pain, 15 cases of traumatic injury and 49 cases of gradual-onset injury were reported. The overall incidence rate of shoulder injuries, lower back injuries and knee injuries was 0.38 injuries (95% CI: 0.30–0.46) per 1000 h of badminton training. The incidence rates of shoulder injuries, lower back injuries and knee injuries were 0.05 injuries (95% CI 0.02–0.07), 0.08injuries (95% CI 0.05–0.12) and 0.25 injuries (95% CI 0.19–0.31) per 1000 h of badminton training (Fig. 2).

Basic parameters of the 460 elementary school age badminton players who were screened from the 510 elementary school age badminton players for pain analysis are shown in Table 1.

**Table 1:** Baseline characteristics of elementary school age badminton players.

Variables	Median (IQR)	N (%)	Pain sites		
			Shoulder (n = 41)	Lower back (n = 32)	Knee (n = 61)
**Gender**					
Male		194 (42.2)	20 (48.8)	14 (43.8)	29 (47.5)
Female		266 (57.8)	21 (51.2)	18 (56.3)	32 (52.5)
**Age (years)**	10.0 (9.0, 11.0)				
7–8		76 (16.5)	10 (24.4)	8 (25.0)	9 (14.8)
9–10		190 (41.3)	12 (29.3)	11 (34.4)	28 (45.9)
11–12		194 (42.2)	19 (46.3)	13 (40.6)	24 (39.3)
Body mass index	16.0 (14.3, 17.8)				
**Experience (year)**	2.3 (1.3, 3.5)				
< 1		81 (17.6)	7 (17.1)	5 (15.6)	12 (19.7)
1 to < 3		210 (45.7)	24 (58.5)	12 (37.5)	27 (44.3)
≥ 3		169 (36.7)	10 (24.4)	15 (46.9)	22 (36.1)
**Hours, per day**	2.5 (2.0, 3.0)				
≤ 2.5		232 (50.4)	15 (36.6)	16 (50.0)	29 (47.5)
> 2.5		228 (49.6)	26 (63.4)	16 (50.0)	32 (52.5)
**Days, per week**	4 (3.0, 5.0)				
≤ 3		128 (27.8)	10 (24.4)	5 (15.6)	12 (19.7)
4–5		247 (53.7)	21 (51.2)	21 (65.6)	36 (59.0)
6–7		85 (18.5)	10 (24.4)	6 (18.8)	13 (21.3)
**Total hours, weekly**	11.3 (8.0, 15.0)				
≤ 11		219 (47.6)	17 (41.5)	15 (46.9)	28 (45.9)
> 11		241 (52.4)	24 (58.5)	17 (53.1)	33 (54.1)
**Warm-up**					
Yes		449 (97.6)	41(100.0)	31 (96.9)	60 (98.4)
No		11 (2.4)	0 (0.0)	1 (3.1)	1 (1.6)
**Cool down**					
Yes		300 (65.2)	29 (70.7)	10 (31.3)	36 (59.0)
No		160 (34.8)	12 (29.3)	22 (68.7)	25 (41.0)

### Association between shoulder pain, lower back pain and knee pain

Out of 611 elementary school age badminton players, 460 players including 194 boys and 266 girls had no experiences of badminton injuries. Results of crude and multivariate logistic regression analysis model for association between shoulder pain, lower back pain and knee pain are shown in Tables 2 and 3. The presence of shoulder pain was significantly associated with knee pain (OR 4.10, 95% CI 2.01–8.38, *p* < 0.001; adjusted OR 4.73, 95% CI 2.18–10.24, *p* < 0.001). The presence of knee pain was significantly associated with lower back pain (OR 3.36, 95% CI 1.51–7.50, *p* = 0.003; adjusted OR 3.72, 95% CI 1.59–8.72, *p* = 0.003), and the presence of lower back pain was significantly associated with shoulder pain (OR 8.26, 95% CI 3.68–18.54, *p* < 0.001; adjusted OR 9.87, 95% CI 4.02–24.21, *p* < 0.001).

**Table 2:** Association between knee pain and shoulder pain among elementary school age badminton players.

Pain sites	Shoulder pain		
	Absence (n = 419)	Presence (n = 41)	*p* value
**Knee pain (n = 61)**			
N (%)	47 (11.2)	14 (34.1)	
OR (95% CI)a	1.00	4.10 (2.01–8.38)	< 0.001
Adjusted OR (95% CI)b	1.00	4.73 (2.18–10.24)	< 0.001

*OR* odds ratio, *CI* confidence intervals.

^a^Crude model.

^b^Adjusted for gender (male or female), age (7–8, 9–10 or 11–12 years), BMI (body mass index), badminton experience (< 1, 1 to < 3 or ≥ 3 years), h per day (≤ 2.5, or > 2.5 h), days per week (≤ 3, 4–5, or 6–7 days), total h per week (≤ 11, or > 11 h), warming up (yes or no) and cool down (yes or no).

**Table 3:** Lower back pain associated with knee pain and shoulder pain among elementary school age badminton players.

Pain sites	Lower back pain (n = 32)		*p* value
	Absence (n = 428)	Presence (n = 32)	
**Knee pain (n = 61)**			
N (%)	51 (12.2)	10 (31.3)	
OR (95% CI)a	1.00	3.36 (1.51–7.50)	0.003
Adjusted OR (95% CI)b	1.00	3.72 (1.59–8.72)	0.003
**Shoulder pain (n = 41)**			
N (%)	29 (6.9)	12 (37.5)	
OR (95% CI)a	1.00	8.26 (3.68–18.54)	< 0.001
Adjusted OR (95% CI)b	1.00	9.87 (4.02–24.21)	< 0.001

*OR* odds ratio, *CI* confidence intervals.

^a^Crude model.

bAdjusted for gender (male or female), age (7–8, 9–10 or 11–12 years), BMI (body mass index), badminton experience (< 1, 1 to < 3 or ≥ 3 years), hours per day (≤ 2.5, or > 2.5 h), days per week (≤ 3, 4–5, or 6–7 days), total hours per week (≤ 11, or > 11 h), warming up (yes or no) and cool down (yes or no).

### Risk factors for shoulder pain

Adjusted OR and 95% CI for shoulder pain using multivariate logistic regression analysis of all the variables are shown in Table 4. Training hours per day was significantly associated with shoulder pain that elementary school-aged badminton players with training time per day > 2.5 h were 2.64 times more likely to sustain shoulder pain than those with training time per day ≤ 2.5 h (hours, per day > 2.5 h: adjusted OR 2.64, 95% CI 1.03–6.78, *p* = 0.043). Gender, age, BMI, badminton experience, days per week, total hours per week, warm-up and cool down were not significantly associated with shoulder pain.

**Table 4:** Adjusted odds ratio for shoulder pain by multivariate analysis.

Variables	Shoulder pain N (%)	Adjusted OR (95% CI)a	*p *value
**Gender**			
Male	20 (48.8)	1.00	
Female	21 (51.2)	1.28 (0.66–2.49)	0.46
**Age (years)**			
7–8	10 (24.4)	1.00	
9–10	12 (29.3)	0.54 (0.21–1.39)	0.20
11–12	19 (46.3)	1.24 (0.44–3.45)	0.69
**Body mass index**			
(per 0.1 increase)	0.91 (0.79–1.05)	0.21	
**Experience (year)**			
< 1	7 (17.1)	1.00	
1 to < 3	24 (58.5)	1.51 (0.59–3.89)	0.40
≥ 3	10 (24.4)	0.58 (0.19–1.77)	0.34
**Hours, per day**			
≤ 2.5	15 (36.6)	1.00	
> 2.5	26 (63.4)	2.64 (1.03–6.78)	0.043
**Days, weekly**			
≤ 3	10 (24.4)	1.00	
4–5	21 (51.2)	1.84 (0.65–5.23)	0.25
6–7	10 (24.4)	3.67 (0.85–15.81)	0.08
**Total hours, weekly**			
≤ 11	17 (41.5)	1.00	
> 11	24 (58.5)	0.47 (0.14–1.57)	0.22
**Warm-up**			
Yes	41 (100.0)	–	–
No	0 (0.0)	–	–
**Cool down**			
Yes	29 (70.7)	1.00	
No	12 (29.3)	1.26 (0.61–2.62)	0.53

*OR* odds ratio, *CI* confidence intervals.

*Significantly associated with shoulder pain, P-value < 0.05.

^a^Adjusted for gender (male or female), age (7–8, 9–10 or 11–12 years), BMI (body mass index), badminton experience (< 1, 1 to < 3 or ≥ 3 years), h per day (≤ 2.5, or > 2.5 h), days per week (≤ 3, 4–5, or 6–7 days), total h per week (≤ 11, or > 11 h), warming up (yes or no) and cool down (yes or no).

### Further analysis of risk factors

In order to identify whether overuse training caused the pain or not, we divided participants into two groups using 2.5 h per day (the risk factor of shoulder pain) as a cut-off point and compared the associations of lower back pain, shoulder pain and knee pain in two groups. The results are shown in Table 5. From the results, there was a significant association between shoulder pain and knee pain as well as between lower back pain and knee pain upon exceeding 2.5 training hours per day. In contrast, whether training hour per day ≤ 2.5 h or > 2.5 h, the presence of lower back pain was always significantly associated with shoulder pain. Among elementary school-aged badminton players with training time per day ≤ 2.5 h, players with lower back pain were 10.50 times more likely to sustain shoulder pain than those without lower back pain, and among those with training time per day > 2.5 h, players with lower back pain were 12.04 times more likely to sustain shoulder pain than those without lower back pain (≤ 2.5 h: adjusted OR 10.50, 95% CI 2.55–43.17, *p* = 0.001; > 2.5 h: adjusted OR 12.04, 95% CI 3.24–44.78, *p* < 0.001, respectively).

**Table 5:** Association between lumber pain, knee pain and shoulder pain broken down by 2.5 h per day.

Pain sites	Training time per day ≤ 2.5 h					
	Knee pain			Shoulder pain		
	Absence (n = 203)	Presence (n = 29)	*p* value	Absence (n = 217)	Presence (n = 15)	*p* value
**Lower back pain (n = 16)**						
N (%)	11 (5.4)	5 (17.2)	11 (5.1)	5 (33.3)		
Adjusted OR (95% CI)a	1.00	2.89 (0.80–10.51)	0.11	1.00	10.50 (2.55–43.17)	0.001
**Shoulder pain (n = 15)**						
N (%)	11 (5.4)	4 (13.8)				
Adjusted OR (95% CI)a	1.00	3.73 (0.94–14.86)	0.06			

	Training time per day > 2.5 h					
	Knee pain			Shoulder pain		
	Absence (n = 196)	Presence (n = 32)	*p *value	Absence (n = 202)	Presence (n = 26)	*p *value
**Lower back pain (n = 16)**						
N (%)	11 (5.6)	5 (15.6)	9 (4.5)	7 (26.9)		
Adjusted OR (95% CI)a	1.00	4.25 (1.24–14.52)	0.021	1.00	12.04 (3.24–44.78)	< 0.001
**Shoulder pain (n = 26)**						
N (%)	16 (8.2)	10 (31.3)				
Adjusted OR (95% CI)a	1.00	5.57 (2.08–14.93)	0.001			

*OR* odds ratio,*CI* confidence intervals.

^a^Adjusted for gender (male or female), age (7–8, 9–10 or 11–12 years), BMI (body mass index), badminton experience (< 1, 1 to < 3 or ≥ 3 years), h per day (≤ 2.5, or > 2.5 h), days per week (≤ 3, 4–5, or 6–7 days), total h per week (≤ 11, or > 11 h), warming up (yes or no) and cool down (yes or no).

## Discussion

We attempted to identify risk factors for shoulder pain, and the association between shoulder pain, lower back pain and knee pain among elementary school-aged badminton players. This study provided unique findings as follows: (1) with increased training hours, there is a significant association between shoulder pain and knee pain as well as between lower back pain and knee pain, (2) training time over 2.5 h per day is a risk factor for shoulder pain, (3) lower back pain was significantly associated with shoulder pain independent of training time.

Some studies of overhead players revealed age^13,22^, gender^23^, training hours per week^14^, training days per week^23^, history of shoulder pain^14^ and training intensity^13^ were risk factors for shoulder pain. Conversely, other studies showed training hours per week as well as training days per week were not associated with shoulder pain^13^. In this study, neither training hours per week nor age showed significant association with shoulder pain. We demonstrated training hours > 2.5 h per day being significantly associated with shoulder pain that elementary school-aged badminton players with training time per day > 2.5 h were 2.64 times more likely to sustain shoulder pain than those with training time per day ≤ 2.5 h. Previous studies of high-level tennis players demonstrated that high values of acute workload (total workload of all training sessions and matches that take place during one week) were associated with risk of injuries^19^ that workload strategy should be monitored. We have not evaluated the training workload in our studies, but the elementary school-aged badminton players with shoulder pain showed more training workload than those without. Thus, although most motor practices could improve motor learning^24^, we suggest players limit training hours to less than 2.5 h per day. On the other hand, although age, gender, height, weight and BMI have been reported to be associated with lower back pain among children and adolescents aged 6–18 years^25,26^, no such association was found in elementary school-aged badminton players of this study.

Shoulder, lower back and knee pains and injuries are frequent among badminton players aged 7–57 years. Among all injuries, shoulder injuries are approximately 1.4–8.7%^1,2,9^, lower back/spine injuries are approximately 1.8–13.7%^1–3,9^, and knee injuries are approximately 10.9–16.2%^1,2,9^, respectively. In this study, 3.0% of injuries associated with badminton are shoulder (50% traumatic injuries and 50% gradual-onset injuries), 5.3% are lower back (38.1% traumatic injuries and 61.9% gradual-onset injuries) and 16.2% are knee (23.4% traumatic injuries and 76.6% gradual-onset injuries) which are in line with previous studies. In respect to injury rate, a retrospective study of badminton injuries reported that injury incidence rates were 0.50 shoulder injuries, 0.34 back injuries and 0.59 knee injuries per 1000 h of badminton playing in elite junior badminton players aged 16–21 years^3^. In other studies of soccer, 0.03 shoulder injuries, 0.01 lower back injuries, 0.20 knee injuries per 1000 h of soccer training^27^ in soccer players aged 7–12 years. In this study, injury rates per 1000 h of badminton training were 0.05 injuries in shoulder, 0.08 injuries in lower back and 0.25 injuries in knee, significantly lower than previous badminton study aged 16–21 years.

With regard to association between shoulder pain, lower back pain and knee pain, there were some studies on baseball, soccer, wrestling and basketball. Some studies of baseball players revealed significant association between shoulder pain and lower back pain, shoulder pain and knee pain, and lower back pain and knee pain^13,14,28^. A previous study of soccer players^29^ also revealed lower back pain was significantly associated with knee pain. Another study of top wrestlers^30^ revealed pain in spine being associated with pain in shoulder and knee. A previous study also revealed that upper extremity pain was significantly associated with lower back pain in elementary and middle school age basketball players^31^. Regarding badminton, a previous study on overhead motion sports including a small number of 95 badminton players revealed shoulder pain in significant association with back pain^14^. In this study, in players with more training hours, shoulder pain was more significantly associated with lower back pain and knee pain, and lower back pain was more significantly associated with knee pain. The findings of our studies agree with previous studies mentioned above.

Weight shifting, balance, joint coordination (i.e., shoulder adduction/abduction, trunk rotation)^32^ and footwork (lunge, jump, crossover stepping)^33,34^ are required during badminton. For forehand overhead stroke motion, trunk is a major segment of overhead motion kinetic chain in transferring energy from lower limbs to upper limbs that contributes to more than 50% of total energy whereas shoulder does 13% of the work^16,35^. Also, in order to generate force, transfer core body mass and maintain balance, it is essential for knee to perform a large movement frequently. During hitting a shuttlecock, several events occur simultaneously including weight shifting, and upper limb and trunk rotation^36^. Therefore, repetitively inadequate motions not only have negative effects on badminton performance, but also cause pains and injuries^32^. The findings of previous studies supported the results of this study that shoulder pain is associated with lower back pain and knee pain, and lower back pain is associated with knee pain in elementary school age badminton players.

However, as training time increases, it is indubitable that pain will occur which may lead to non-related shoulder pain, lower back pain and knee pain as a result. Additionally, players who feel pain more easily may also be more likely to feel pain in multiple sites, but these pains may not be related as well. In other words, we cannot determine the associations between pains of two different sites unless we exclude the effects of training time and pain threshold. To do so, we divided the participants into two groups by training hours. Interestingly, when training hours per day was taken into consideration, in training hours per day ≤ 2.5 h group, a significant association was found between lower back pain and shoulder pain whereas no significant association were found between lower back pain and knee pain, shoulder pain and knee pain. In training hours per day > 2.5 h group, there were significant association between shoulder pain, lower back pain and knee pain, respectively. In other words, lower back pain was associated with shoulder pain independent of training time while shoulder pain was associated with knee pain, lower back pain was associated with knee pain only when training time > 2.5 h per day. The results are not all in line with previous studies where knee pain was associated with lower back pain and shoulder pain^13,14,28–31^.

Lower back which weaken the ability of the lower back acting as an energy transmitter, can cause improper trunk rotation. This will result in greater maximal shoulder external rotation angle that accordingly alter shoulder joint loading^37^. In other words, lower back pain creates an intermittent load that has to be compensated for by movement of shoulder which causes shoulder pain. Our result that lower back pain is associated with shoulder pain independent of training time support the statement. To help preventing shoulder pain, training time per day and lower back pain should be taken into consideration by coaches and elementary school age badminton players. Also, coaches, physicians and physiotherapists should pay attention to potential injury risks in knee as well as trunk and shoulder when players’ training time > 2.5 h per day.

In baseball, youth pitchers exhibit many risky behaviors which are associated with increased likelihood of pitching with shoulder tiredness and shoulder pain. Therefore, the American Sports Medicine Institute made pitching guidelines (e.g., pitch count limits and required rest recommendations) based on decades of research for baseball players aged-22 years to prevent shoulder injury^38^. Furthermore, it also appeals to each baseball organization for establishing rules to ensure that players must follow the guidelines while training. Badminton is a popular overhead motion racket sport played by more than 220 million people^39^, however, no such playing guidelines were made for youth badminton players. In this study some risk factors were identified, and we believe other risk factors will be detected and age-appropriate playing guidelines for badminton players will be designed in future.

There are some limitations in the current study. First, the study is retrospective, cross-sectional instead of prospective, so it was unclear whether the association was causal. Second, the intensities of pain in this study were not investigated. Thirdly, motion performance, such as very high-speed running may increase injury risk in training^20^. Even though motion performance has been reported to be a significant role for preventing badminton pains and injuries, we did not investigate action performed (e.g., overhead motion strokes, lunge, jumping and direction changes) of the participants during the training. Finally, intrinsic factors such as physical fitness and badminton motor skills were not investigated in this study. Shoulder range of motion (e.g., glenohumeral internal rotation deficit, external rotation gain, total rotation loss)^40,41^, general joint laxity^42,43^, hamstring tightness^44^, core stability^45^ and trunk rotation^46–48^ have been revealed in association with body pain or injuries. Improper motion skills were regarded as mechanism of body pain or injuries associated with overhead motion sports^16,32,46,47,49^. Thus, the studies of physical fitness and overhead motion skills in elementary school-aged badminton players will be the next target.

## Conclusions

This study is the first to identify risk factors for shoulder pain and associations of pain at different anatomical sites in elementary school badminton players aged 7–12 years. Training hours per day is a strong risk factor for shoulder pain which should be restricted to ≤ 2.5 h per day. Lower back pain, shoulder pain and knee pain were associated with each other, respectively. Moreover, the findings stated that lower back pain was significantly associated with shoulder pain independent of training time. For preventing badminton pains, appropriate training programme should be implemented and the complaints of the lower limbs as well as trunk and upper limbs should be noticed.
